# Fabrication of a Heptapeptide-Modified Poly(glycidyl Methac-Rylate) Nanosphere for Oriented Antibody Immobilization and Immunoassay

**DOI:** 10.3390/molecules29194635

**Published:** 2024-09-29

**Authors:** Xiaoxing Gong, Jie Zhang, Liyan Zhu, Shu Bai, Linling Yu, Yan Sun

**Affiliations:** 1 Department of Biochemical Engineering, School of Chemical Engineering and Technology, Tianjin University, Tianjin 300350, China; gongxiaoxing@tju.edu.cn (X.G.); zhangjie94@tju.edu.cn (J.Z.); lyzhu@tju.edu.cn (L.Z.); sbai@tju.edu.cn (S.B.); 2 Key Laboratory of Systems Bioengineering and Frontiers Science Center for Synthetic Biology (Ministry of Education), Tianjin University, Tianjin 300350, China

**Keywords:** oriented antibody immobilization, antigen recognition, affinity binding peptide, poly(glyceryl methacrylate) nanospheres, adsorption thermodynamics, activity

## Abstract

Oriented antibody immobilization has been widely employed in immunoassays and immunodiagnoses due to its efficacy in identifying target antigens. Herein, a heptapeptide ligand, HWRGWVC (HC7), was coupled to poly(glycidyl methacrylate) (PGMA) nanospheres (PGMA-HC7). The antibody immobilization behavior and antigen recognition performance were investigated and compared with those on PGMA nanospheres by nonspecific adsorption and covalent coupling via carbodiimide chemistry. The antibodies tested included bovine, rabbit, and human immunoglobulin G (IgG), while the antigens included horseradish peroxidase (HRP) and β-2-Microglobulin (β2-MG). The nanospheres were characterized using zeta potential and particle size analyzers, scanning electron microscopy, transmission electron microscopy, Fourier transform infrared spectroscopy, and reversed-phase chromatography, proving each synthesis step was succeeded. Isothermal titration calorimetry assay demonstrated the strong affinity interaction between IgG and PGMA-HC7. Notably, PGMA-HC7 achieved rapid and extremely high IgG adsorption capacity (~3 mg/mg) within 5 min via a specific recognition via HC7 without nonspecific interactions. Moreover, the activities of immobilized anti-HRP and anti-β2-MG antibodies obtained via affinity binding were 1.5-fold and 2-fold higher than those of their covalent coupling counterparts. Further, the oriented-immobilized anti-β2-MG antibody on PGMA-HC7 exhibited excellent performance in antigen recognition with a linear detection range of 0–5.3 μg/mL, proving its great potential in immunoassay applications.

## 1. Introduction

Antibody immobilization onto solid surfaces with high activity and capacity is generally required for the identification and detection of target antigens from complex biological samples in immunoassays, immunodiagnoses, and immunoseparation [1,2,3,4,5]. Among the various strategies for antibody immobilization, oriented immobilization has been regarded as one of the most attractive strategies [6] due to its advantage in maintaining the effective activity of antibodies and maintaining high antigen-binding capacity [7,8]. 

Generally, most oriented immobilization strategies are based on selectively noncovalent bio-affinity binding or specific covalent reactions only at specific sites [9]. For the former, protein A/G–immunoglobulin G (IgG) [10] and streptavidin–biotin [11] are the typical cases. Meanwhile, for the latter, the click chemistries serve as the representatives [12]. In comparison, the peptide ligands of small molecules with high affinity to the antibodies would be more economical and robust for large-scale oriented antibody immobilization because they are easily synthesized by well-established methods and feature high robustness [13,14,15,16,17].

**Figure 1:**
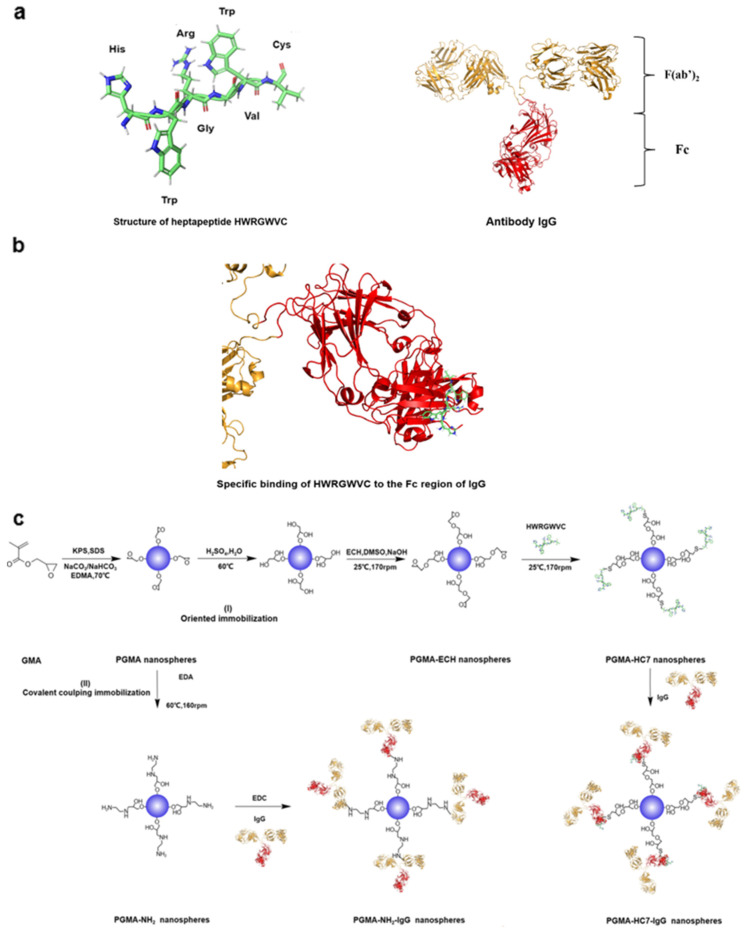
Schematic diagram of (**a**) heptapeptide HWRGWVC and IgG, (**b**) specific identification on the Fc region of IgG by HWRGWVC, and (**c**) synthesis routes of PGMA nanospheres for IgG immobilization. (I) PGMA-HC7 nanospheres for specific binding via HWRGWVC; (II) PGMA-NH_2_ nanospheres for EDC-based covalent immobilization of IgG.

Nowadays, numerous synthetic peptides are designed to specifically recognize and bind the conserved fragment crystallizable (Fc) region of IgG [18,19], which is one of the most widely utilized types of antibodies in bioanalytical analysis [20]. The Fc binding peptides made excellent exposure of the fragment antigen binding (Fab) region of IgG and thus maintained an easy approachability to the antigen-binding sites of the immobilized antibodies [21]. For instance, the hexapeptide ligand HWRGWV, which is composed of a typical N-terminal histidine residue followed by an aromatic amino acid residue (W) and positively charged amino acid residue (R), could specifically recognize the Fc region of the antibody, with an Fc selectivity comparable to protein A [17,21]. Additionally, its derivative, the heptapeptide HWRGWVC (HC7), which features a C-terminal cysteine residue for convenient coupling (Figure 1a), has been shown to specifically bind to the Fc region (Figure 1b), demonstrating an affinity for IgG that is two orders of magnitude stronger than that of the original HWRGWV in our previous research [22]. Moreover, HC7-modified nonporous poly(glycidyl methacrylate) (PGMA) microspheres significantly enhanced the capacity and activity for the oriented immobilization of IgG [22]. However, only a simple model antigen–antibody pair, consisting of horseradish peroxidase (HRP) and anti-HRP IgG, has been tested on the HC7-modified PGMA microspheres. The potential for antigen recognition related to human diseases remains untested, leaving their applicability in detecting clinical biomarkers uncertain. Furthermore, the limited specific surface area of the nonporous PGMA microspheres as a solid support restricts their effectiveness in actual antigen recognition. 

In contrast, nanoparticles with favorable specific surface areas are more suitable and have been successfully applied in immunoassays [23,24,25,26]. Although nanoparticles of various components have been reported [27,28,29,30,31], PGMA nanosphere is one of the most prominent materials in immunoassays due to its ease of preparation, low cost, and easy modification by a simple ring-opening reaction [32,33,34]. Therefore, in this study, HC7 was covalently coupled to the surface of PGMA nanospheres (~100 nm) for the oriented immobilization of antibodies in order to systematically explore the immunoassay performance, and the oriented antibody immobilization behaviors, including thermodynamics, kinetics, capacity, affinity, activity, and the detection range of immobilized antibodies on HC7-modified nanospheres, were investigated. The bovine IgG was used as a model antibody in the binding thermodynamic and kinetic experiments. In addition to the model antigen–antibody pair of HRP rabbit anti-HRP IgG, β-2-microglobulin (β2-MG), a typical biomarker related to human diseases and widely used in immunoassays and clinical diagnostics, was selected as the representative antigen, with the human anti-β2-MG IgG serving as the representative antibody, in order to evaluate the practical application potential in immunoassays. Additionally, immobilizing antibodies on the PGMA nanospheres via passive adsorption and covalent coupling by carbodiimide chemistry was also performed as the control (Figure 1). As far as we know, this is the first report on the immunoassay/immunodiagnose system based on HC7-modified PGMA nanosphere, and the experimental results are expected to contribute to the development of cost-effective and efficient immunoassay systems for practical applications.

## 2. Results and Discussion

### 2.1. Characterization of PGMA Nanospheres

**Figure 2:**
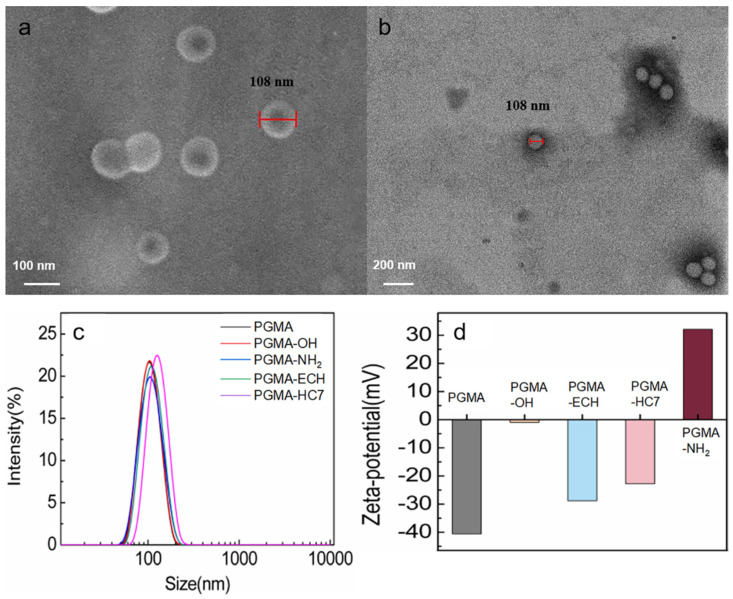
The SEM and TEM images of PGMA-HC7 nanospheres. (**a**) SEM at magnifications of 500 nm and (**b**) TEM at magnifications of 200 nm. Size distributions (**c**) and Zeta potentials (**d**) of different nanospheres.

Mono-sized PGMA nanospheres and their derivatives were successfully synthesized, as clearly illustrated in Figure 2 and Figure S1. The physical properties of the PGMA-based nanospheres produced in this study are detailed in Table 1. The FTIR spectra of these PGMA-based nanospheres (Figure S1) align with our previous findings on PGMA-based microspheres [22], proving the successful synthesis. In detail, the PGMA nanospheres (Figure S1a) identified their specific groups, including the 848 and 908 cm^−1^ for epoxy group asymmetrical stretching vibration and 1720 cm^−1^ for C=O of ester group stretching vibration. For PGMA-OH and PGMA-NH_2_ nanospheres (Figure S1b,c), the characteristic vibrations at 3420 and 1037 cm^−1^ and 3361 and 1560 cm^−1^ appeared, respectively, while the vibrations at 848 and 908 cm^−1^ disappeared, demonstrating that the hydroxyl and amino groups were successfully introduced into the PGMA nanospheres through the modification of the epoxy groups. 

The morphology of the PGMA-HC7 nanospheres, as shown in Figure 2a,b, indicates that the nanospheres exhibit well-defined spherical shapes. They all fall within a narrow size distribution range with a mean particle size of 108 nm (Figure 2a,b), which corresponds with the result of the size analysis presented in Figure 2c and Table 1. The average diameter of the PGMA-based nanospheres tested in aqueous solution was 100–120 nm with a narrow size distribution and a low PDI value (0.02–0.05), proving the surface modifications did not present significant effects on the particle size (Figure 2c and Table 1). The few differences in their particle sizes were attributed to the differences in swelling and hydrophilic properties after surface modification, which were clearly presented in Figure 2d. Specifically, PGMA nanospheres with many epoxy groups showed a surface potential of −40.6 mV. After opening the epoxy rings, the surface potential of PGMA-OH nanospheres with high hydrophilicity was close to 0 mV. Further modification into PGMA-NH_2_, the surface potential changed to positive. The PGMA-ECH nanospheres also have many epoxy groups on the surface, so the surface potential of −28.8 mV was observed. For the PGMA-HC7 nanospheres, the coupling density of HC7 peptide was adjusted by the added amount of HWRGWVC in the coupling reaction, and the complete disappear peak at 7.5 min in the chromatograms (Figure S2) indicated the full coupling of 60 μmol ligand per g dry nanospheres (Table 1).

### 2.2. Thermodynamic Analysis of IgG Binding

**Figure 3:**
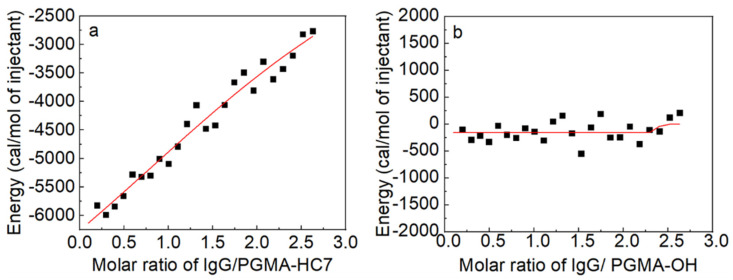
ITC isotherms of IgG binding to (**a**) PGMA-HC7 and (**b**) PGMA-OH nanospheres.

The ITC assay was conducted to investigate the binding interactions between PGMA-HC7 nanospheres and IgG, with the PGMA-OH nanospheres serving as a control (Figure 3). As illustrated in Figure 3a, the binding of IgG to PGMA-HC7 was an exothermic reaction, evidenced by significant changes in enthalpy (ΔH < 0), which indicates a strong adsorption interaction between HC7 and IgG. However, the heat changes for IgG binding to PGMA-OH were very small, as shown in Figure 3b, proving the weak binding between them. Because the molar ratio of IgG to PGMA-HC7 higher than 3 would induce the aggregation of PGMA-HC7 nanospheres, which would affect the calorimetry, the fitting of thermodynamic parameters, including the changes in enthalpy (ΔH), entropy (ΔS), and Gibbs free energy (ΔG), were not attempted. Nevertheless, the data presented in Figure 3a indicate that the binding constant (*K*_b_) between PGMA-HC7 and IgG was 10^5^~10^6^ M^−1^, which conformed to affinity adsorption (the value of Kb was in the range of 10^4^~10^8^ M^−1^) [15]. Additionally, the strong affinity of PGMA-HC7 to IgG agreed with our previous study on the HC7-modified PGMA microspheres, whose binding constant for IgG was 1~6 × 10^6^ M^−1^ [22]. These results confirmed that the peptide ligand HC7 serves as an effective tool for the specific capture of IgG.

### 2.3. Batch Adsorption Behaviors of IgG onto PGMA Nanospheres

**Figure 4:**
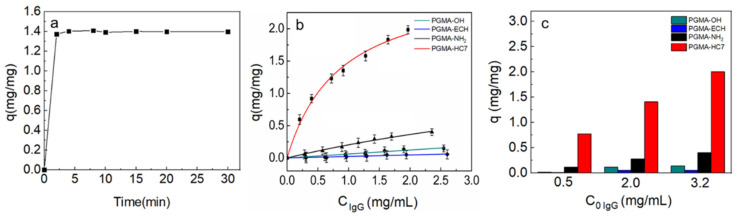
(**a**) Uptake kinetic curve of 2 mg/mL bovine IgG on PGMA-HC7 nanospheres in physiological buffer at 19,000 rpm. (**b**) Adsorption isotherms of bovine IgG on different PGMA-based nanospheres. (**c**) Adsorption density of bovine IgG on the PGMA-based nanospheres at varying initial concentrations.

To investigate the antibody capture information of the heptapeptide HWRGWV-modified PGMA nanospheres, bovine IgG adsorption kinetics and equilibria were investigated with the same methods described in our previous work on PGMA microspheres [22]. Meanwhile, other PGMA nanospheres without coupling HC7, including PGMA-OH, PGMA-ECH, and PGMA-NH_2_ nanospheres, were used for comparisons. The static adsorption results are provided in Figure 4, and the fitted dissociation constant (*K*_d_) and maximum capacity (*q*_m_) are summarized in Table 1.

Figure 4a demonstrates that the adsorption of bovine IgG onto PGMA-HC7 nanospheres was quick, and the adsorption equilibrium was reached within 3 min due to the simple surface adsorption onto the nonporous PGMA nanospheres with little resistance to mass transfer surface adsorption onto the nonporous PGMA nanospheres [35,36]. After reaching equilibrium, the adsorbed IgG amount kept the same value with increasing time from 3 min to 30 min (Figure 4a). Therefore, in the subsequent adsorption equilibria and immobilization experiments, the adsorption time was set to 5 min. It is noteworthy that the general time required for immobilizing antibodies is ~0.5 h [16], and our previous work on HC7-modified PGMA microspheres also required a 0.5 h immobilization [22]. The much less time for immobilization in this work would be beneficial for immunoassays, proving the significant advantage of the PGMA-HC7 nanospheres.

The adsorption isotherms presented in Figure 4b indicate that a typical Langmuir adsorption isotherm was observed for the PGMA-HC7 nanospheres. In contrast, linear adsorption isotherms were obtained for the other PGMA-based nanospheres without coupling HC7 (PGMA-OH, PGMA-ECH, and PGMA-NH_2_), which was caused by their differences in binding interactions. Obviously, IgG binding on the PGMA-HC7 nanospheres is based on specific bio-affinity interaction between the peptide HC7 and IgG [18], as shown in Figure 1b. However, the IgG binding on the other nanospheres is probably caused by weak, nonspecific interactions. For instance, hydrophobic binding by the epoxy groups [37] or electrostatic binding by the amino groups [38]. Because of the weak nonspecific binding, much lower adsorption densities were obtained for the three PGMA-based nanospheres without coupling HC7. Consequently, the Langmuir equation was not suitable to describe their isotherms [22,39,40,41]. To better compare the immobilization capacity of these nanospheres and consider the generally fixed concentrations used in practical immobilization processes for immunoassays, the adsorption density at the same intimal IgG concentration for these nanospheres is given in Figure 4c. The far higher adsorption densities of PGMA-HC7 than other PGMA-based nanospheres (approximately 5–40 folds) were exhibited in Figure 4b,c, demonstrating the stronger IgG capture ability with negligible nonspecific interactions of PGMA-HC7 nanospheres. 

It is worth noting that the fitted *q*_m_ value for PGMA-HC7 nanospheres was 2.76 mg/mg, and that of HC7-modified microspheres was only 2.44–3.33 mg/g [22], as listed in Table 2. That is, PGMA-HC7 nanospheres achieved about 1000-fold higher IgG binding capacity than HC7-modified microspheres. The extremely high IgG binding capacity of PGMA-HC7 nanospheres proved its superiority in antibody immobilization and further indicated the nonporous PGMA-based nanospheres would perform higher efficiency in antigen recognition over nonporous PGMA-based microspheres, which would be investigated in the following section. The fitted *K*_d_ value for PGMA-HC7 nanospheres (5.8 × 10^−6^ M) was similar to other HC7-based materials reported in the literature [42,43] but a little bit larger than our previous HC7-modified microspheres [22], as listed in Table 2. The little difference in binding affinity was considered due to the differences in IgG sources and peptide coupling densities. Overall, the above results proved the obvious superiority of PGMA-HC7 nanospheres in specific IgG immobilization, including the fast uptake equilibrium, extremely high immobilization capacity, and favorably strong affinity. 

### 2.4. Oriented Antibody Immobilization Performance of PGMA-HC7 Nanospheres

**Figure 5:**
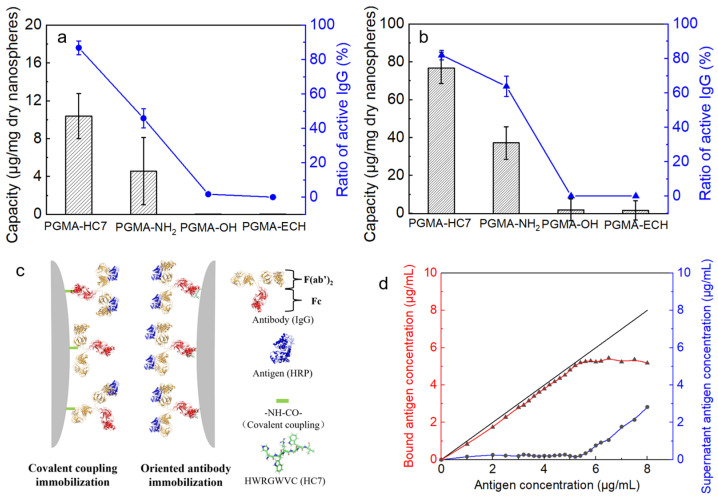
Antibody immobilizations onto different PGMA-modified nanospheres. (**a**) Capacity and activity of immobilized anti-HRP IgG. (**b**) Capacity and activity of immobilized anti-β2-MG IgG. (**c**) Schematic drawing of the covalent coupling and orientated immobilization of IgG. (**d**) Detection range of PGMA-HC7-anti-β2-MG-IgG system.

The activity of immobilized IgG serves as the primary evaluation indicator for immobilization efficiency in practical applications, i.e., antigen recognition based on specific binding between antigens and antibodies. Therefore, in this section, the antigen–antibody pairs of HRP and rabbit anti-HRP IgG and β2-MG and human anti-β2-MG IgG were utilized to explore the immobilization performance of PGMA-HC7 nanospheres, including the capacity and activity. IgG immobilization by the passive binding on PGMA-OH and PGMA-ECH and via the EDC-based covalent linking on pGMA-NH_2_ were performed for comparison. The immobilization results are provided in Figure 5.

It is obvious in Figure 5a,b that after the washing for the removal of the non-specifically immobilized IgG, the immobilization capacity of PGMA-OH and PGMA-ECH is negligible, and no active antibody has been detected on them. The results further confirmed that all the IgG molecules on PGMA-HC7 were specifically bound by HC7 without nonspecific interactions. Moreover, due to a new adsorption–desorption equilibrium being reached with the physiological buffer in the washing process, the amounts of two immobilized IgGs on the PGMA-HC7 nanospheres were much lower than that in Figure 4. Nevertheless, the immobilization capacity of affinity binding (PGMA-HC7) for these two antibodies was over 2-fold higher than that of covalent coupling (PGMA-NH_2_), and the active antibody on PGMA-HC7 was 1.7 to 2.7 folds higher than that on PGMA-NH_2_. In addition, the ratio of active antibody (active antibody/total antibody immobilized) on different nanospheres was also calculated, and active ratios of PGMA-HC7 were nearly 1.3 to 2 folds higher than those of PGMA-NH_2_ (86.8% vs. 45.8% and 81.9% vs. 63.7%, respectively, in Figure 5a,b), proving the advantages of orientated immobilization over the covalent coupling (Figure 5c). These results also verified the universality of the PGMA-HC7 nanospheres in antibody immobilization for practical immunoassay applications.

It is noteworthy for the immobilization of rabbit anti-HRP IgG and human anti-β2-MG IgG, PGMA-HC7 nanospheres achieved 10–80 ug/mg dry nanospheres (Figure 5a,b), but the HC7-modified PGMA microspheres only reached ~30 ng/mg dry microspheres for anti-HRP IgG immobilization [22]. In other words, the immobilization capacity of the PGMA-HC7 nanospheres was 1000-fold higher than that of the HC7-modified PGMA microspheres (ug/mg dry nanospheres vs. ng/mg dry microspheres). Moreover, the ratio of active antibodies of the former was 10-fold higher than that of the latter (~80% vs. 7%) [22]. These results further corroborated that the nonporous PGMA-based nanospheres performed higher efficiency in antigen recognition over nonporous PGMA-based microspheres. Additionally, both the immobilization capacity and the ratio of active antibody on PGMA-HC7 nanospheres were much higher than those on other HWRGWVC-linked nanoparticles [29], whose immobilization capacity and the ratio of active antibody were only about 1.5 ug/mg and 50%, respectively [27,29,44].

Furthermore, considering the well-known risk of dissociation of affinity-based (noncovalent) antibody immobilization from the solid support, we also checked the residual amount of active antibody on the PGMA-HC7 nanospheres after several washes (n ≥ 3). It was found that both the total amounts of the immobilized antibody and the amounts of the active antibody on the PGMA-HC7 nanospheres reduced after these washes. Nevertheless, the ratio of active antibodies increased to 100% after these washes, proving that PGMA-HC7 nanospheres performed well in the maintenance of IgG bioactivity during their use process. By contrast, the PGMA-NH_2_ nanospheres kept an active ratio of ~50%. That is, the amount of active antibody on the PGMA-HC7 nanospheres kept 2-fold higher than the covalent coupling (pGMA-NH_2_), demonstrating the stability of the formed PGMA-HC7-IgG system and its potential for immunoassay applications. Then, the detection range of the PGMA-HC7-anti-β2-MG-IgG system was determined by adding the varying concentrations of β2-MG (Figure 5d), and it is clear that almost no antigen was detected in the supernatant at β2-MG concentrations of ≤5.3 μg/mL, indicating that the antigen β2-MG was completely bound to the PGMA-HC7-anti-β2-MG-IgG system. In other words, the recognized antigen amount of the PGMA-HC7-anti-β2-MG-IgG system exhibited a linear relationship with the added antigen amount in the detection system in the β2-MG concentration range of 0–5.3 μg/mL. This detection range is comparable to or even wider than other antigen detection systems based on HC7 or other affinity peptides [27,29,44], proving the PGMA-HC7-anti-β2-MG-IgG system is promising in immunoassay applications.

It is known that HC7 was derived from the HWRGWV peptide that has a Fc selectivity comparable to protein A but not comparable to protein G [15,16,17], and HC7 showed two orders of magnitude stronger affinity for IgG than its original hexapeptide ligand HWRGWV [22]. Thus, it could be deduced that the IgG selectivity of HC7 would be comparable to protein A/G. Considering the much lower cost and higher stability of the short peptide ligand than the macromolecular to protein A/G, the PGMA-HC7 nanospheres are more practicable and economical for practical application in antibody immobilization.

The above results proved that there are four main advantages of this PGMA-HC7 nanospheres over other antibody immobilization techniques: (1) Rapid immobilization. PGMA-HC7 nanospheres achieved rapid adsorption equilibrium for IgG immobilization at only 3 min (Figure 4a). Hence, the immobilization time was set to 5 min in this work, while the general time required for immobilizing antibodies is ~0.5 h [16,22]. (2) High capacity. An extremely high adsorption capacity of ~3 mg/mg could be achieved by the specific binding of antibodies on PGMA-HC7 nanospheres within 5 min (Table 2), which is much higher than other nanomaterials [22]. Specifically, other HC7-modified nanospheres exhibited a capacity of only 1.5 μg/mg [27]. In addition, the adsorption capacity achieved in this work was 1000-fold higher than that of HC7-modified microspheres in our previous work [22]. (3) High activity. The ratio of active antibodies on the PGMA-HC7 nanospheres achieved ~90% for both rabbit anti-HRP IgG and human anti-β2-MG IgG (Figure 5a,b). It is not only 1.5–2 times higher than that on the covalent immobilization on PGMA nanospheres but also higher than other HC7-modified nanospheres and microspheres. For instance, only 7% of active antibodies were observed on HC7-modified microspheres in our previous work [22], and only 50% was reported for other HC7-modified nanospheres [29]. (4) Economical preparation. PGMA nanospheres with characteristics of ease of preparation, low cost, and easy modification by a simple ring-opening reaction, and the HC7 peptide ligand with characteristics of high affinity to IgG, lower cost, and high stability, afforded the PGMA-HC7 nanosphere to be one of the most practicable, economical, and prominent strategies in practical antibody immobilization.

However, this technique based on PGMA-HC7 nanospheres also has limitations. For instance, PGMA-HC7 nanospheres could only be used for binding antibodies with an Fc region, but the reorganization of other types of antibodies without an Fc region is not applicable. In particular, nanobodies with small size, simple structure, high affinity, and remarkable stability [45,46], which has become one of the most attractive antibodies currently, do not have the Fc region (Figure S3). Therefore, further efforts on the reorganization of nanobodies are needed.

In short, the practical potentials of PGMA-HC7 nanospheres in immunoassay applications have been demonstrated, not only for their economical preparation, stable and strong affinity, and low cost, but also for their fast and high capacity in antibody immobilization with a wide linear detection range and high sensitivity.

## 3. Materials and Methods

### 3.1. Materials

Glycidyl methacrylate (GMA) and ethylene glycol dimethacrylate (EGDMA) were purchased from Alfa Aesar (Shanghai, China). Bovine IgG was purchased from GL Biochem (Shanghai, China). The peptide HWRGWVC (HC7, 95% purity) was synthesized by GL Biochem Ltd. (Shanghai, China). Rabbit anti-HRP IgG (1 mg/mL) and ELISA kits for anti-rabbit IgG were from Biosynthesis Biotechnology Co., Ltd. (Beijing, China). β2-MG, human anti-β2-MG IgG, and their analysis kits were purchased from Epsilon Biotechnology Co., Ltd. (Zhejiang, China). Dimethyl sulfoxide (DMSO) and epichlorohydrin (ECH) were of analytical grade from the Guangfu Fine Chemical Research Institute (Tianjin, China). Potassium persulfate (KPS), HRP, 3,3′,5,5′-tetramethylbenzidine (TMB), HRP-TMB kits, and other reagents were from Sangon Biotech Co., Ltd. (Shanghai, China).

### 3.2. Fabrication of Mono-Sized PGMA Nanospheres

The mono-sized PGMA nanospheres were synthesized according to the previous method of emulsion-polymerization [32,47,48] with slight modifications. Briefly, 20 mM Na_2_CO_3_, 20 mM NaHCO_3_, 0.2% (*w*/*w*) potassium persulfate, 0.5% (*w*/*v*) sodium dodecyl sulfate, and 10% GMA were mixed together, and 10 mL of the mixture was incubated under a nitrogen atmosphere in an air bath under shaking (100 rpm) at 25 °C for 15 min to start the emulsification reaction. After adding 0.2% EGDMA (cross-linker), the polymerization reaction was contained in a water bath at 70 °C and 180 rpm for 12 h. Finally, the PGMA nanospheres were obtained by dialysis with deionized water to remove the impurities at room temperature and concentrated in PEG 20000; the concentration of each nanosphere suspension was confirmed by the previous method [22].

### 3.3. Surface Modification of PGMA Nanospheres

The nanospheres with different surface modifications were prepared in accordance with the undermentioned steps, and the reaction routes are provided in Figure 1.

Firstly, 5 mL PGMA nanosphere suspension (7.5 mg dry nanospheres/mL) was added to 10 mL 0.5 M H_2_SO_4_ solution and then placed in a water bath at 160 rpm and 60 °C for 3 h to open the epoxy groups into hydroxyl groups [49], and the product was labeled as PGMA-OH.

Secondly, 5 mL PGMA-OH nanospheres suspension (8.7 mg dry nanospheres/mL) was mixed with 2 mL ECH, 4 mL NaOH (1 M), and 4 mL DMSO and then placed in a water bath at 25 °C and 170 rpm for 2 h, and the product was denoted as PGMA-ECH.

Then, 5 mg HWRGWVC was mixed with 20 mL PGMA-ECH nanosphere suspension (4.4 mg dry nanospheres/mL) and 20 mL phosphate buffer, following the same method as the previous work [22]. Finally, in order to hydrolyze the residual epoxy groups of nanospheres, the excess NaBH_4_ was added, and the product was labeled as PGMA-HC7. 

In addition, 5 mL PGMA nanosphere suspension (7.5 mg dry nanospheres/mL) was mixed with 5 mL deionized water and 20 mL EDA at 170 rpm and 70 °C for 12 h, and the product was denoted as PGMA-NH_2_ for the covalent coupling of IgG.

After each modification step, the nanospheres were purified by dialysis with deionized water and concentrated in PEG 20000.

### 3.4. Characterization of PGMA Nanospheres 

The average diameter and zeta potential of PGMA nanospheres with different surface modifications were measured by ZetaSizer Nano ZS Malvern Instruments (Worcestershire, UK), and the polydispersity index (PDI) was measured to represent the particle size distribution. The microscopic morphologies, size, and homogeneity of the nanospheres were observed by scanning electron microscopy (SEM, JEOL Ltd., Tokyo, Japan) and transmission electron microscopy (TEM, Tecnai G2 F20, FEI, Hillsboro, OR, USA). To characterize the structure of the nanospheres, Fourier transform infrared spectroscopy (FTIR, Bio-Rad, Hercules, CA, USA) was employed. The peptide ligand density of the PGMA-HC7 nanospheres was calculated by the same method as previous work [22]. 

All measurements mentioned above, average diameter, zeta potential, PDI, and peptide density, were measured at least three times for each sample (in triplicate).

### 3.5. Isothermal Titration Calorimetry (ITC) Assay

In order to determine the binding affinity between PGMA-HC7 nanospheres and IgG, the free energy of IgG binding to PGMA-HC7 nanospheres was determined by an ITC assay (VP-ITC, MicroCal, Amherst, MA, USA). Briefly, a drop of the IgG solution (139 µM) was injected into the PGMA-HC7 suspension (with the HC7 of 10 µM), and 25 injections with 8-min intervals were performed for each titration. All titration experiments were conducted at 37 °C. The PGMA-OH nanospheres were used as the control. The titration data were used to fit the parameters of the binding phenomenon to calculate the binding constant (Kb) [50].

### 3.6. Batch Binding Experiments

The bovine IgG was selected as the model antibody to investigate the batch adsorption behavior of antibodies on different surface-modified nanospheres (PGMA-OH, PGMA-ECH, PGMA-NH_2_, and PGMA-HC7) under physiological conditions of the physiological buffer at 37 °C.

Firstly, 1 mL bovine IgG solution of 2 mg/mL was added to 2 mL PGMA-HC7 nanosphere suspension, respectively, and incubated at 37 °C for 1–30 min to investigate the uptake kinetics. After each incubation, the supernatant was separated by centrifugation for 30 min at 19,000 rpm, and its IgG concentration was determined by spectrophotometry at λ = 280 nm. The binding density (q, mg-IgG/mg dry nanosphere) of bovine IgG onto PGMA-HC7 nanospheres varying with time was calculated by mass balance.

Then, the 1 mL bovine IgG solution with different concentrations of 0.4 mg/mL, 0.8 mg/mL, 1.2 mg/mL, 1.6 mg/mL, 2 mg/mL, 2.4 mg/mL, 3.2 mg/mL, and 4 mg/mL were added to the 2 mL nanospheres suspension, respectively, and incubated at 37 °C for 5 min to obtain the adsorption isotherms. The Langmuir isotherm model was used to describe the adsorption isotherm data as,

*q* = *q*_m_*c*/(*K*_d_ + *c*), (1)

where *c* is the IgG concentration in the supernatant after adsorption equilibrium (mg/mL), *K*_d_ is the dissociation constant (mg/mL), *q* is the adsorption density (mg-IgG/mg dry nanosphere), and *q*_m_ is the maximum capacity obtained by fitting (mg-IgG/mg dry nanosphere). Three parallel tests were performed in each group, and the fitted *q*_m_ and *K*_d_ were informed of their standard errors.

### 3.7. Antibody Immobilization for Antigen Recognition

Human anti-β2-MG IgG and rabbit anti-HRP IgG were immobilized onto PGMA-HC7 nanospheres under physiological conditions to explore the antigen recognition performance using HRP and β2-MG, respectively.

For immobilization by specific binding, 2 mL PGMA-HC7 nanosphere suspension was mixed with 1 mL anti-β2-MG IgG or anti-HRP IgG solution (2 mg/mL) at 37 °C for 5 min and then separated by high-speed refrigerated centrifugation at 19,000 rpm for 30 min. The concentration of residual IgG in the supernatant was detected by kits to calculate the amount of immobilized IgG using mass balance. Additionally, the saved PGMA-HC7 nanospheres (after immobilizing IgG, denoted as PGMA-HC7-IgG) were cleaned with 1 mL physiological buffer to remove the nonspecific adsorbed IgG and separated by centrifugation at 19,000 rpm for 30 min, and the concentration of residual IgG in the washing supernatant was detected to calculate the amount of IgG specifically bound to the PGMA-HC7 nanospheres (after washing once) by mass balance. 

For the analysis of the activity of the immobilized IgG on PGMA-HC7, the 300 μL antigen solution (β2-MG or HRP 100 μg/mL) was added to the above collected PGMA-HC7-IgG nanospheres (after washing once) and incubated at 37 °C for 10 min, and then centrifuged at 19,000 rpm for 30 min. The amount of antigen in the separated supernatant was determined by the quantitative kits, and the amount of antigen linked with the immobilized IgG was calculated by mass balance, which was used to represent the amount of active immobilized IgG after washing once. Additionally, the beads with IgG and antigen were cleaned with a physiological buffer, and the remaining activity of IgG on the nanospheres was calculated.

For the determination of the antigen detection range of the PGMA-HC7-IgG system, the antigen–antibody pair of β2-MG and anti-β2-MG IgG were used. Briefly, antigen β2-MG solution with different concentrations was added to the equal volume of PGMA-HC7-IgG nanospheres (after washing once) suspension, respectively, and incubated for 10 min at 37 °C. Then, the mixture was separated by centrifugation at 19,000 rpm for 30 min, and the amount of residual antigen in the supernatant was detected by the quantitative kit.

The antibody immobilization onto nanospheres without HC7 (PGMA-OH, PGMA-ECH, and PGMA-NH_2_) was also conducted for comparisons. Three parallel experiments were carried out for each measurement mentioned above, and the average value was represented by its standard deviation.

## 4. Conclusions

In this work, the affinity-binding peptide HWRGWVC was modified to PGMA nanospheres to investigate its role in the oriented immobilization of antibodies for immunoassay applications. It is worth noting that PGMA-HC7 achieved an exceptionally high IgG binding capacity of ~3 mg/g within 5 min via strong specific binding (*K*_d_ value of 5.8 × 10^−6^ M) of HC7 peptide without nonspecific interactions. While other HC7-free PGMA nanospheres only presented a much lower capacity for bovine IgG binding, approximately one-fifth to one-tenth of that of PGMA-HC7. Moreover, the feasibility, effectiveness, and universality of PGMA-HC7 in antibody binding and antigen recognition were verified by the evidence of both higher immobilization capacity and ratio of active antibody by affinity binding of rabbit anti-HRP IgG and human anti-β2-MG IgG, comparing with covalent coupling of PGMA-NH_2_ via EDC and nonspecific binding of PGMA-OH and PGMA-ECH. Particularly, PGMA-HC7 demonstrated an active IgG ratio exceeding 90%, confirming its advantages in oriented antibody immobilization. Further, the formed PGMA-HC7-anti-β2-MG-IgG system exhibited a linear detection range of 0–5.3 μg/mL for β2-MG recognition, demonstrating the practical potential of PGMA-HC7 nanospheres in immunoassay applications. The results provide an easy, low-cost, but stable way to prepare carriers affording rapid, strong affinity and high capacity for high-active antibody immobilization and antigen recognition systems with a wide linear detection range and high sensitivity. In our subsequent work, we will direct towards the detection of the actual samples using the HC7-modified nanospheres and microspheres. 

## Figures and Tables

**Table 1:** Physical properties of the PGMA-based nanospheres.

Nanosphere	Average Size (nm)	PDI	HC7 Density (μmol/g Dry Nanospheres)
PGMA	102.6	0.033	0
PGMA-OH	107.3	0.027	0
PGMA-ECH	110.1	0.039	0
PGMA-HC7	119.3	0.021	60
PGMA-NH_2_	108.8	0.049	0

**Table 2:** Adsorption parameters of the HC7-modified nanospheres and microspheres.

Immobilization Carrier		Equilibrium Time (min)	*q*_m_ (mg/mg)	*K*_d_ (mg/mL)
Nanosphere	PGMA-HC7	5	2.76 ± 0.16	0.87 ± 0.12
Microsphere	pGMA-HC7-25 ^a^	30	(3.33 ± 0.28) × 10^−3^	0.13 ± 0.06
	pGMA-HCH-75 ^a^	30	(2.44 ± 0.10) × 10^−3^	0.03 ± 0.01

^a^: data from Ref. [22].

## Data Availability

Data are available on request from the authors.

## Supplementary Materials

The following supporting information can be downloaded at: https://www.mdpi.com/article/10.3390/molecules29194635/s1, Figure S1: FTIR spectra of (a) PGMA, (b) PGMA-OH and (c) PGMA-NH2 nanospheres; Figure S2: Reversion phase liquid chromatograms of the reaction solution before and after HWRGWVC coupling; Figure S3: Structure of the nanobody (a) and IgG (b).
