# Supplementary material for: Fabrication of a Heptapeptide-Modified Poly(glycidyl Methac-Rylate) Nanosphere for Oriented Antibody Immobilization and Immunoassay
Source: Molecules. 2024 Sep 29;29(19):4635. doi: 10.3390/molecules29194635 (PMC11477792; doi:10.3390/molecules29194635)
Supplement: Supplementary file 1 [file molecules-29-04635-s001.zip › molecules-3175725-supplementary.pdf]

Supplementary Materials

Fabrication of a Heptapeptide-Modified Poly(glycidyl Methac-Rylate) Nanosphere for Oriented Antibody Immobilization and Immunoassay

Xiaoxing Gong [1], Jie Zhang [1], Liyan Zhu [1], Shu Bai [1,2], Linling Yu [1,2,*] and Yan Sun [1,2,*]

[1] Department of Biochemical Engineering, School of Chemical Engineering and Technology, Tianjin University, Tianjin 300350, China; gongxiaoxing@tju.edu.cn (X.G.); zhangjie94@tju.edu.cn (J.Z.); lyzhu@tju.edu.cn (L.Z.); sbai@tju.edu.cn (S.B.)
[2] Key Laboratory of Systems Bioengineering and Frontiers Science Center for Synthetic Biology (Ministry of Education), Tianjin University, Tianjin 300350, China
* Correspondence: yulinling@tju.edu.cn (L.Y.); ysun@tju.edu.cn (Y.S.)

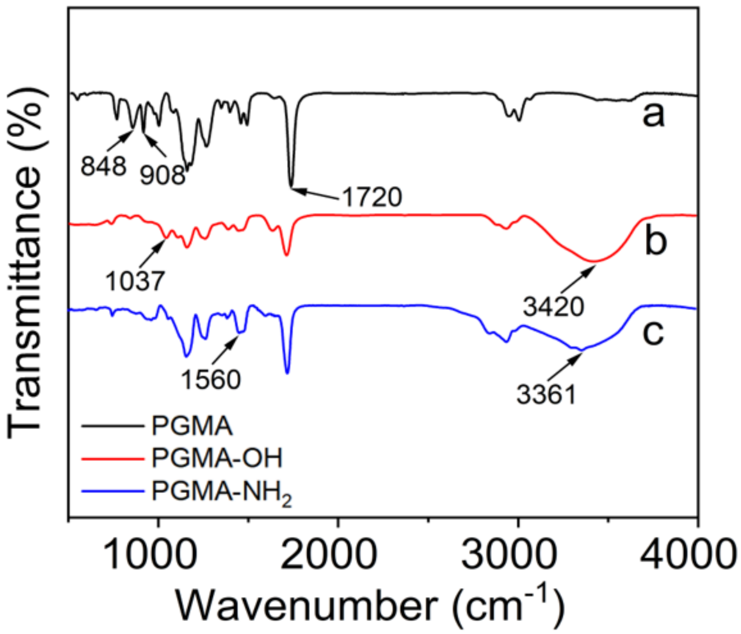

Figure S1. FTIR spectra of (a) PGMA, (b) PGMA-OH and (c) PGMA-NH_2 nanospheres.

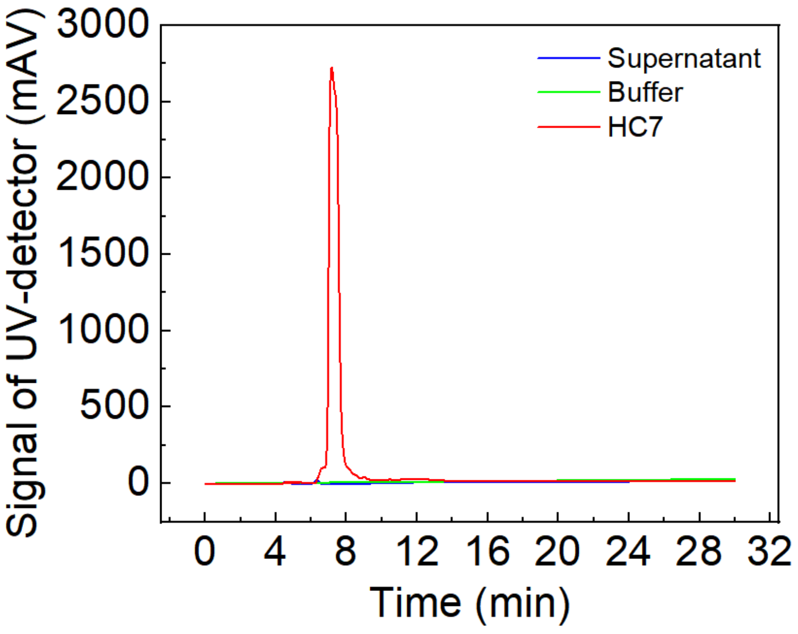

Figure S2. Reversion phase liquid chromatograms of the reaction solution before and after HWRGWVC coupling.

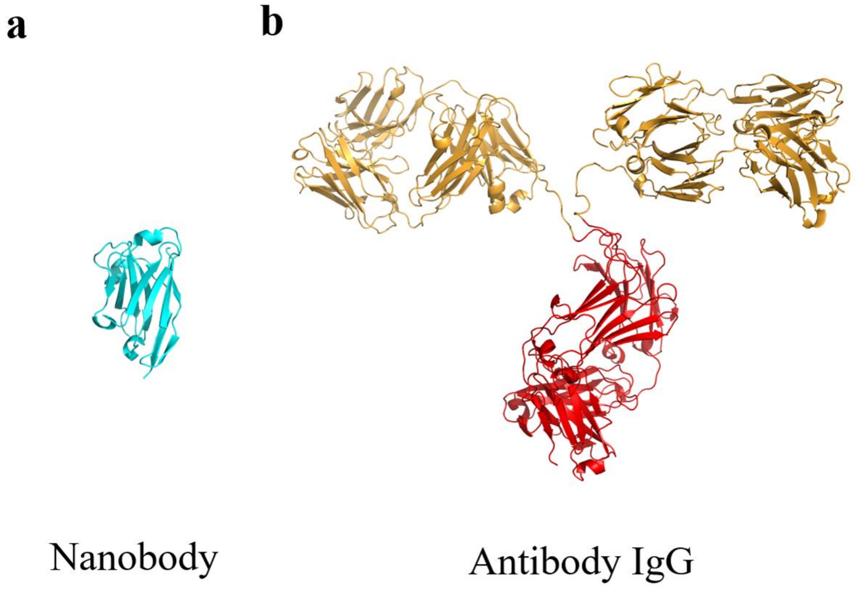

Figure S3. Structure of the nanobody (a) and IgG (b).
